# The Role of Methylmalonic Acid in the Risk of Sarcopenia and All‐Cause Mortality Among Individuals With Sarcopenia: Evidence From NHANES

**DOI:** 10.1002/fsn3.70841

**Published:** 2025-09-03

**Authors:** Ping Zhu, Jie Zhang, Xue‐Chun Liu, Ming Song, Bin Lu, Zhi‐Cheng Yang, Hui Pan, Ya‐Qiong Jiao, Ya‐Fei Guo, Fang‐Fang Chen, Zhi‐Hao Wang, Bo‐Ang Hu, Ming Zhong

**Affiliations:** ^1^ State Key Laboratory for Innovation and Transformation of Luobing Theory; Key Laboratory of Cardiovascular Remodeling and Function Research, Chinese Ministry of Education, Chinese National Health Commission and Chinese Academy of Medical Sciences; Department of Cardiology Qilu Hospital of Shandong University Jinan China; ^2^ Department of Cardiology People's Hospital of Lixia District of Jinan Jinan China; ^3^ Department of Zero‐Magnetic Medicine Qilu Hospital of Shandong University Jinan China; ^4^ School of Nursing and Rehabilitation, Cheeloo College of Medicine Shandong University Jinan China; ^5^ Department of Geriatric Medicine & Laboratory of Gerontology and Anti‐Aging Research Qilu Hospital of Shandong University Jinan China; ^6^ Department of Life Science Imperial College London UK; ^7^ Department of Cardiology The First Affiliated Hospital of Shandong First Medical University & Shandong Provincial Qianfoshan Hospital Jinan China

**Keywords:** methylmalonic acid, mortality, NHANES, sarcopenia, vitamin B12 deficiency

## Abstract

Mitochondrial dysfunction is increasingly recognized as a driver of sarcopenia pathogenesis, progression, and prognosis. Muscle mass is a fundamental and objective component of sarcopenia. In some studies, relative muscle loss has been used to define sarcopenia. Methylmalonic acid (MMA) is a biomarker of mitochondrial dysfunction and vitamin B12 deficiency. Evidence has shown a negative link between MMA and muscle function, yet population‐level evidence on its predictive and prognostic value in low muscle mass and sarcopenia remains scarce. This cohort study analyzes 10,414 U.S. adults from the National Health and Nutrition Examination Survey. Incidence of low muscle mass is evaluated with adjusted logistic regression, while all‐cause mortality risk in this population is assessed using Cox regression and Kaplan–Meier analysis. Restricted cubic splines (RCS) model MMA‐mortality dose–response. Subgroup and sensitivity analyses test robustness. After full covariate adjustment, elevated MMA level independently predicts low muscle mass incidence (OR = 1.30, 95% CI: 1.08–1.56, *p* = 0.007) and all‐cause mortality (HR = 2.17, 95% CI: 1.64–2.89, *p* < 0.001) in this population. RCS analysis demonstrates a monotonic mortality increase with rising MMA concentrations (*p* for overall < 0.001), with no evidence of nonlinearity (*p* for nonlinear = 0.057). Kaplan–Meier survival curve exhibits significant mortality divergence across MMA tertiles (log‐rank *p* < 0.001), especially in the elder low muscle mass population. Subgroup analysis identifies higher mortality associations in lifetime alcohol abstainers (HR = 3.60, 95% CI: 2.34–5.53, *p* < 0.001) and diabetic/borderline populations (HR = 3.49, 95% CI: 2.35–5.20, *p* < 0.001) with low muscle mass. Notably, MMA has significant interaction effects with congestive heart failure (*p* for interaction = 0.002). Sensitivity analysis corroborates the robustness of these associations. Serum MMA could serve as a dual biomarker for independently predicting low muscle mass incidence and post‐diagnosis mortality. These findings underscore the clinical utility for early risk detection and prognosis stratification as well as call for trials targeting MMA reduction to mitigate sarcopenia pathogenesis, progression, and prognosis.

## Graphical abstract

In this U.S. cohort of 10,414 adults, elevated serum methylmalonic acid (MMA) level independently predicted a 30% increased risk of developing sarcopenia. Among sarcopenic individuals, higher MMA was strongly associated with a more than 2‐fold increase in all‐cause mortality risk, with risks rising progressively as MMA concentrations increased. Serum MMA aids in predicting sarcopenia incidence and stratifying prognosis after diagnosis.

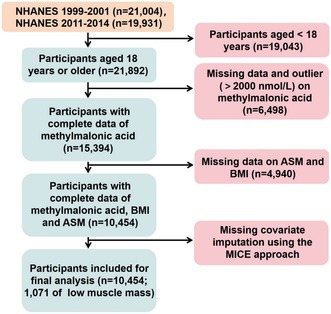

## 1 Introduction

Sarcopenia, identified as a systemic and progressive skeletal muscle disorder, manifests through diminished muscle mass, reduced strength, and/or decreased physical performance. It is particularly prevalent among older adults. The reported prevalence of sarcopenia ranges from 5% to 50% (Patel et al. 2020; Shafiee et al. 2017). Significantly, sarcopenia is independently associated with a 60% increase in all‐cause mortality among community‐dwelling older people and even 86% among nursing home residents (Liu et al. 2017; Benz et al. 2024; Zhang et al. 2018). According to the major international consensus documents released in 2019 (EWGSOP2 and AWGS 2019), reduced muscle strength has been formally established as the core essential criterion for diagnosing sarcopenia. However, reliance on late‐stage functional decline for sarcopenia diagnosis fails to capture early muscle quality deterioration and predict patient prognosis (Chao et al. 2025; Gupta et al. 2024). Consequently, relative muscle loss has been used to define sarcopenia in some studies (Batsis et al. 2016; Huang et al. 2024). Compelling evidence has revealed mitochondrial dysfunction and oxidative stress are vital pathological mechanisms underlying sarcopenia pathogenesis, progression, and prognosis (Habets et al. 2022; Kim et al. 2023; Li et al. 2022; Xu et al. 2025; Zhang et al. 2023). Disrupted energy metabolism and abnormal reactive oxygen species (ROS) levels process before clinically detectable muscle mass loss and continue throughout the pathological process (Kemp et al. 2020; Kunzke et al. 2020; Marques et al. 2023; Sha et al. 2023). The mechanistic understanding highlights the mitochondrial dysfunction biomarkers could surpass conventional diagnostic modes and enable early‐stage risk stratification and precise prediction of adverse outcomes.

Methylmalonic acid (MMA) is a key intermediate in mitochondrial metabolism. Under physiological conditions, MMA is enzymatically converted to succinic acid for integration into the tricarboxylic acid (TCA) cycle (Green and Miller 2022; Hannibal et al. 2016). This conversion requires functional mitochondrial methylmalonyl‐CoA mutase and adequate vitamin B12 (cobalamin) levels, with deficiencies in either component leading to pathological MMA accumulation (Green and Miller 2022). The accumulation is highly specific to metabolic pathways and differs from other mitochondrial‐related drivers of sarcopenia, such as inflammatory responses or age‐related mitochondrial functional degradation. Although the latter two significantly promote the progression of sarcopenia, they do not directly raise circulating MMA level. Moreover, it is worth noting that increasing findings evidence a key role of supernumerary MMA in mitochondrial dysfunction and oxidative stress, by compromising the mitochondrial respiratory chain, reducing ATP content, and inducing reactive oxygen species (ROS) generation (Guo et al. 2024; Chandler et al. 2009; Chu et al. 2019; Stepien et al. 2017; Weidemann et al. 1970). Clinically, comprehensive adjustment models in elderly cohorts demonstrate persistent associations between elevated MMA and musculoskeletal deterioration, independent of confounding factors including vitamin B12 (Wolffenbuttel et al. 2020; Zhao et al. 2024).

Notably, prospective cohort studies establish MMA as an independent mortality predictor across populations and a prognostic indicator for chronic diseases (Riphagen et al. 2020; Wang et al. 2020). An elevated risk of all‐cause mortality by 33% per unit increase in log‐transformed MMA is observed (Wang et al. 2020). Similar elevation emerges in diabetes, cancers, cardiovascular, and chronic kidney disease populations, even after full adjustment for potential covariates (Wang, Tang, et al. 2022; Wang, Wang, et al. 2022; Liu et al. 2024; Dhar et al. 2023; Guo et al. 2023; Wang et al. 2020; Zhu et al. 2023; Wu et al. 2023). While elevated MMA demonstrates robust associations with mortality in most populations, its specific prognostic value in sarcopenia individuals remains contentious. Contemporary investigations predominantly focus on delineating the association between MMA and sarcopenia, whereas longitudinal cohort studies examining MMA's temporal relationship with sarcopenia‐specific mortality are conspicuously absent (Wolffenbuttel et al. 2020; Zhao et al. 2024). Consequently, further large cohort studies are needed.

In this study, we leveraged data from the National Health and Nutrition Examination Survey (NHANES) to investigate two underexplored relationships: (1) whether serum MMA level predicts incident low muscle mass in adults, and (2) whether serum MMA modulates all‐cause mortality risk in individuals with low muscle mass. Our findings will provide novel evidence elucidating the role of mitochondrial metabolite derangement in sarcopenia progression and prognosis.

## 2 Materials and Methods

### 2.1 Study Population

**FIGURE 1:**
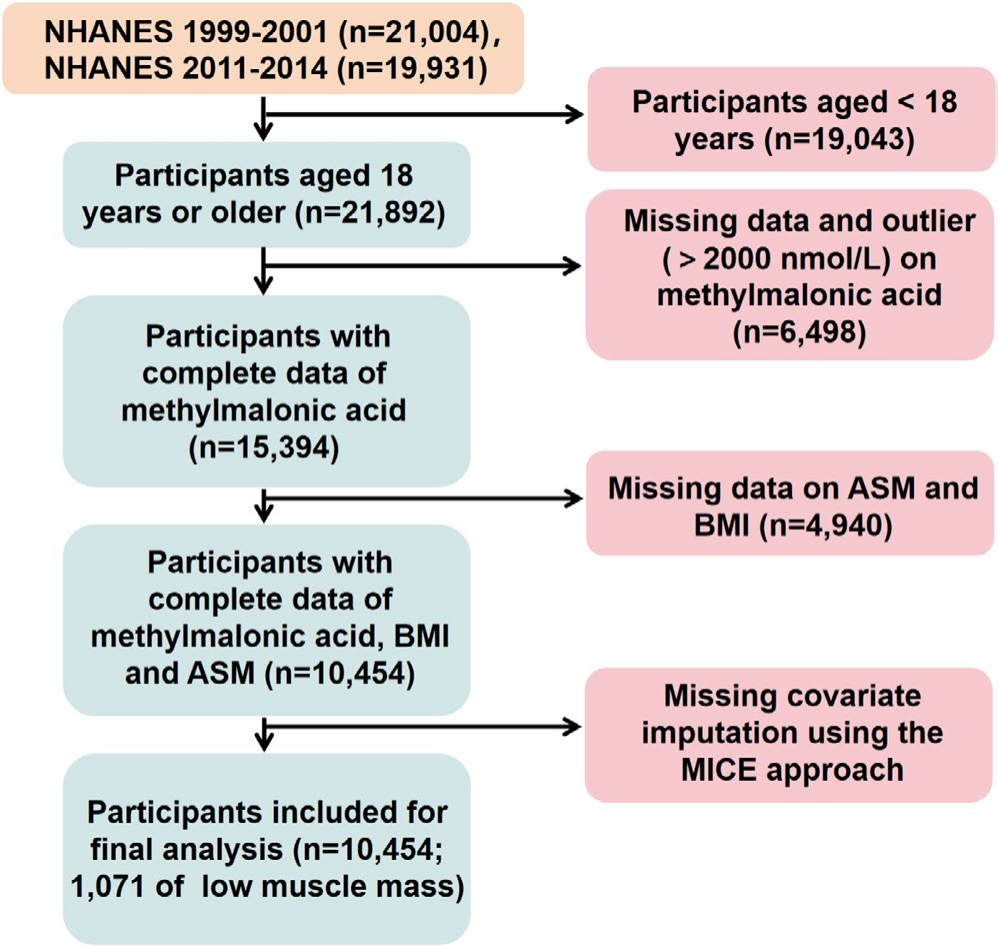
The flow diagram of participants selection from the NHANES 1999 to 2002 and 2011 to 2014. ASM, appendicular skeletal muscle mass; BMI, body mass index; MICE, multiple imputation methods; NHANES, National Health and Nutrition Examination Survey.

This investigation utilized publicly available data from the NHANES, a nationally representative surveillance program jointly administered by the National Institutes of Health (NIH) and the Centers for Disease Control and Prevention (CDC) to monitor health trends and nutritional profiles of U.S. residents. Our analysis focused on aggregated data spanning four survey cycles (1999–2002 and 2011–2014), comprising 40,935 initial participants. As outlined in Figure 1, exclusion criteria involved: (1) individuals under 18 years (*n* = 19,043), (2) cases with unavailable MMA measurements (*n* = 6493), and (3) participants missing appendicular skeletal muscle mass or BMI values (*n* = 4945), resulting in a final analytical sample of 10,454 adults (1071 with low muscle mass). To address incomplete covariate data, we implemented multiple imputation through predictive mean matching algorithms supported by Markov chain Monte Carlo simulations, iterating the process five times to enhance statistical validity and reduce selection bias. All computations were executed in R version 4.4.1 using the “mice” package (v3.16.0) for data imputation and modeling.

### 2.2 MMA Measurement

Serum samples were collected via venipuncture at mobile examination centers (MECs) following NHANES protocols. MMA quantification was performed using two distinct analytical platforms across survey cycles: gas chromatography–mass spectrometry (GC/MS) for the 1999 to 2004 cohorts and isotope‐dilution liquid chromatography–tandem mass spectrometry (LC–MS/MS) for the 2011 to 2014 cohorts. For LC–MS/MS analysis, a 75‐μL serum aliquot was mixed with deuterated internal standard (d3‐MMA), followed by liquid–liquid extraction using tert‐butyl methyl ether. The extracted MMA underwent butanol derivatization to form dibutyl esters, which were then reconstituted in acetonitrile/water. Chromatographic separation and quantification were achieved through multiple reaction monitoring, with MMA concentrations calculated based on the peak area ratios of MMA to d3‐MMA, using the units of nmol/L. Detailed methodological specifications are publicly accessible on the NHANES official website.

### 2.3 Sarcopenia Definition

Sarcopenia diagnosis followed sex‐specific thresholds established by the Foundation for the National Institutes of Health (FNIH) Osteoarthritis Biomarkers Consortium, defined as an appendicular skeletal muscle mass (ASM) to body mass index (BMI) ratio < 0.789 in males and < 0.512 in females (Eckstein et al. 2015; Hunter et al. 2014).

Although functional tests like grip strength and walking speed were common ways to define sarcopenia (Masanés et al. 2017), our study failed to use them because the data were not consistently available. Specifically, the NHANES survey conducted from 1999 to 2002 only included walking speed, whereas grip strength was only recorded during surveys in 2011–2014. To keep our whole dataset comparable, we used a reduction in muscle mass as an indicator of sarcopenia instead (Studenski et al. 2014; Wang et al. 2020). Skeletal muscle mass was a core element of sarcopenia that could be reliably measured. While using muscle mass effectively identified sarcopenia related to mass loss, it might not fully show the physical function problems associated with the disease. Future research including these functional tests would give a more complete picture of sarcopenia.

### 2.4 Covariates

Based on previous epidemiological investigations, we identified covariates potentially influencing the MMA‐sarcopenia/mortality in sarcopenia adult relationship (Huang et al. 2024; Zeng et al. 2024). Race/ethnicity comprised five categories: Mexican American, non‐Hispanic Black, non‐Hispanic White, other Hispanic, and other Race. Educational attainment was grouped into two levels: below high school (encompassing 9th grade or below and 9th–11th grade without diploma) and high school graduate or above (including high school graduate, some college/AA and degree college graduate/above). Family poverty income ratio (PIR) was stratified as ≤ 1.0, 1.0 to 3.00, and > 3.0. Body mass index (BMI) classifications adhered to WHO criteria: underweight (< 18.5 kg/m^2^), normal (18.5–24.9 kg/m^2^), overweight (25.0–29.9 kg/m^2^) and obese (≥ 30.0 kg/m^2^). Participants were dichotomized by age into 18 to 65 years and those aged 65 or older. Smoking behavior was defined using lifetime cigarette exposure: never smokers (< 100 cigarettes in life), former smokers (≥ 100 cigarettes in life but currently quit), and current smokers (≥ 100 cigarettes and ongoing smoking). Alcohol consumption patterns included lifetime abstainers (< 12 alcohol drinks in life), former drinkers (≥ 12 alcohol drinks in life but < 12 in the past year), and current drinkers (≥ 12 alcohol drinks both in life and the past year). Physical activity levels were quantified using metabolic equivalent of task (MET) minutes per week, with inactive (< 600 MET‐min/week), moderate (600–8000 MET‐min/week), and vigorous (≥ 8000 MET‐min/week) categories (Kyu et al. 2016). Missing covariate data were handled through multiple imputation to minimize potential bias.

### 2.5 Statistical Analysis

In the NHANES study, a complex sampling design incorporating sample weights (WTMEC2YR), clustering (SDMVPSU), and stratification (SDMVSTRA) was used. Descriptive statistical analysis was conducted on demographic information. Continuous variables such as vitamin B12 and energy intake were presented as the means ± standard deviation (SD), and categorical variables such as sex and education level were expressed as frequencies (*n*) and percentages (%). To compare the differences between low muscle mass and the control group (or decreased and alive group with low muscle mass), chi‐squared (*χ* ^2^) tests were applied for categorical variables. Given that MMA was significantly skewed, we performed a natural logarithm (ln) transformation on it and categorized them into 3 tertiles (T1, T2, and T3) based on the concentrations in serum.

### 2.6 Statistical Model 1: Weighted Multivariate Regression

We employed weighted multivariate logistic regression models to evaluate the associations of MMA with low muscle mass and weighted multivariate Cox regression models to evaluate the associations of MMA with all‐cause mortality in the low muscle mass population. MMA was utilized as continuous and categorical variables, respectively. When MMA was classified based on its tertile distribution, the first tertile group was considered the reference group. We first assessed the relationship between MMA and low muscle mass or mortality (crude model). Model 1 was subsequently adjusted for demographic data, including sex, race, family PIR, BMI, age, education, and marital status. Based on Model 1, Model 2 was further adjusted for lifestyle, including energy intake, protein intake, smoking, alcohol drinking, and physical activity. Model 3 was further adjusted for comorbidities, including congestive heart failure and diabetes on the basis of Model 2. The results were presented as odds ratios (ORs) or hazard ratios (HRs) and corresponding 95% confidence intervals (CIs). In addition, the weighted restricted cubic spline regression (RCS) was used to model the potential nonlinear association between MMA and all‐cause mortality in low muscle mass adults. The model was conducted with 3 knots of ln MMA.

### 2.7 Statistical Model 2: Kaplan–Meier Survival Curve

Kaplan–Meier survival curve was generated to study the relationship between MMA and all‐cause mortality in low muscle mass adults. Participants were stratified into three equally sized groups (tertiles) based on ln MMA level: T1 (lowest), T2, and T3 (highest).

### 2.8 Statistical Model 3: Sensitivity Analysis

To assess the robustness of the findings, we conducted three separate weighted sensitivity analyses: (1) excluding participants with implausibly low energy intake (< 1000 kcal/day; *n* = 152), (2) removing older adults aged ≥ 65 years (*n* = 330) to minimize age‐related confounding, and (3) eliminating individuals diagnosed with congestive heart failure (*n* = 54) to address potential comorbidity confounding. The same weighted multivariate Cox proportional hazards model was then applied to reanalyze the data in both the crude and adjusted models. All analytic models maintained statistical significance (*p* < 0.05) across these restricted cohorts.

### 2.9 Statistical Model 4: Subgroup Analysis

Subgroup‐specific HRs were estimated through stratified weighted Cox proportional hazards models under the complex survey sampling framework, with covariate adjustment for demographic characteristics (race, education, marital status, family PIR) and lifestyle (energy or protein intake). Multiplicative interactions were formally assessed via likelihood ratio tests comparing models with versus without product terms, with Holm‐Bonferroni correction applied to interaction *p*‐values to account for multiple hypothesis testing (*α* = 0.05, two‐tailed). Stratification variables included: sex (male or female), age (18–65 or ≥ 65 years), BMI categories, lifestyle factors (smoking/drinking status and physical activity intensity), and comorbidity profiles (diabetes and congestive heart failure status).

All statistical analysis and plotting were performed using R software version 4.4.1, and a two‐sided *p* < 0.05 was considered statistically significant.

## 3 Results

### 3.1 Baseline Characteristics

The demographic features of the 10,454 adults from NHANES 1999–2002 and 2011–2014 were presented in Table 1. The cohort was stratified by muscle mass status according to FNIH standards (*n* for low muscle mass = 9383 and *n* for normal muscle mass = 1071). Among them, the group with low muscle mass had a higher proportion of obese participants, accounting for approximately 58.5%. Compared to the normal muscle mass group, participants with low muscle mass were more likely to be Mexican American, obese, younger, married or living with a partner, never‐smokers and current‐drinkers, have higher MMA, lower energy or protein intake, moderate physical activities and higher educational levels, as well as with family PIR > 1 and ≤ 3, but without congestive heart failure and diabetes. Significant differences were observed in these baseline characteristics (*p* < 0.05). More specific details could be seen in Table 1. Weighted baseline characteristics were shown in Table S1.

**TABLE 1:** Baseline characteristics of included participants according to muscle mass status.

Characteristics	Overall (*N* = 10,454)	Normal muscle mass (*N* = 9383)	Low muscle mass (*N* = 1071)	*p*
MMA, nmol/L (mean ± SD)	150.53 ± 104.62	148.24 ± 99.45	170.57 ± 140.57	< 0.001***
Sex, *n* (%)				
Male	5256 (50.3%)	4714 (50.2%)	542 (50.6%)	0.821
Female	5198 (49.7%)	4669 (49.8%)	529 (49.4%)	
Race, *n* (%)				
Mexican American	2106 (20.1%)	1648 (17.6%)	458 (42.8%)	< 0.001***
Non‐Hispanic Black	4247 (40.6%)	3872 (41.3%)	375 (35.0%)	
Non‐Hispanic White	811 (7.8%)	712 (7.6%)	99 (9.2%)	
Other Hispanic	2154 (20.6%)	2102 (22.4%)	52 (4.9%)	
Other Race	1136 (10.9%)	1049 (11.2%)	87 (8.1%)	
Family PIR, *n* (%)				
≤ 1.0	2472 (23.6%)	2152 (22.9%)	320 (29.9%)	< 0.001***
> 1.0, ≤ 3.0	4113 (39.3%)	3632 (38.7%)	481 (44.9%)	
> 3.0	3869 (37.0%)	3599 (38.4%)	270 (25.2%)	
BMI, *n* (%)				
Underweight	190 (1.8%)	184 (2.0%)	6 (0.6%)	< 0.001***
Normal	3337 (31.9%)	3218 (34.3%)	119 (11.1%)	
Overweight	3436 (32.9%)	3116 (33.2%)	320 (29.9%)	
Obese	3491 (33.4%)	2865 (30.5%)	626 (58.5%)	
Age group, *n* (%)				
18–65	9350 (89.4%)	8609 (91.8%)	741 (69.2%)	< 0.001***
≥ 65	1104 (10.6%)	774 (8.2%)	330 (30.8%)	
Education, *n* (%)				
Below high school	2796 (26.7%)	2295 (24.5%)	501 (46.8%)	< 0.001***
High school graduate/above	7658 (73.3%)	7088 (75.5%)	570 (53.2%)	
Marital status, *n* (%)				
Married/living with partner	5942 (56.8%)	5256 (56.0%)	686 (64.1%)	< 0.001***
Widowed/separated/divorced/never married	4512 (43.2%)	4127 (44.0%)	385 (35.9%)	
Folate, ng/mL RBC (mean ± SD)	392.77 ± 192.43	392.10 ± 190.11	398.69 ± 211.70	0.330
Vitamin B12, pg/mL (mean ± SD)	629.22 ± 2743.43	611.91 ± 2005.27	780.86 ± 6184.11	0.374
Energy intake, kcal/day (mean ± SD)	2187.24 ± 1050.35	2229.63 ± 1063.14	1815.86 ± 844.64	< 0.001***
Protein intake, g/day (mean ± SD)	83.19 ± 44.84	84.48 ± 45.34	71.89 ± 38.44	< 0.001***
Smoke, *n* (%)				
Current smoker	2374 (22.7%)	2210 (23.6%)	164 (15.3%)	< 0.001***
Former smoker	2137 (20.4%)	1834 (19.5%)	303 (28.3%)	
Never smoker	5943 (56.8%)	5339 (56.9%)	604 (56.4%)	
Alcohol, *n* (%)				
Current drinker	7540 (72.1%)	6873 (73.2%)	667 (62.3%)	< 0.001***
Former drinker	1413 (13.5%)	1252 (13.3%)	161 (15.0%)	
Lifetime abstainer	1501 (14.4%)	1258 (13.4%)	243 (22.7%)	
Physical activity, *n* (%)				
Inactive	3285 (31.4%)	2875 (30.6%)	410 (38.3%)	< 0.001***
Moderate	6007 (57.5%)	5413 (57.7%)	594 (55.5%)	
Vigorous	1162 (11.1%)	1095 (11.7%)	67 (6.3%)	
Congestive heart failure, *n* (%)				
No	10,268 (98.2%)	9251 (98.6%)	1017 (95.0%)	< 0.001***
Yes	186 (1.8%)	132 (1.4%)	54 (5.0%)	
Diabetes, *n* (%)				
No	9494 (90.8%)	8623 (91.9%)	871 (81.3%)	< 0.001***
Yes	802 (7.7%)	632 (6.7%)	170 (15.9%)	
Borderline	158 (1.5%)	128 (1.4%)	30 (2.8%)	
Survival, *n* (%)				
No	1515 (14.5%)	1156 (12.3%)	359 (33.5%)	< 0.001***
Yes	8939 (85.5%)	8227 (87.7%)	712 (66.5%)	
Follow up, months (mean ± SD)	139.43 ± 74.76	139.68 ± 74.63	137.25 ± 75.85	0.322

Abbreviations: BMI, body mass index; MMA, methylmalonic acid; PIR, poverty income ratio.

*** *p* < 0.001.

Table 2 presented the baseline characteristics of the low muscle mass adults categorized by all‐cause mortality status. The median follow‐up time for the entire cohort was 137 months. Adults in the deceased group (*n* = 359) were more likely to be male, non‐Hispanic Black, obese, older, never smokers, with lower educational, folate/energy/protein intake, and physical activity levels. However, the alive group (*n* = 712) tended to have lower MMA level, congestive heart failure, and diabetes. Weighted baseline characteristics were shown in Table S2.

**TABLE 2:** Baseline characteristics of low muscle mass participants according to mortality status.

Characteristics	Total (*N* = 1071)	Alive (*N* = 712)	Deceased (*N* = 359)	*p*
MMA, nmol/L (mean ± SD)	170.57 ± 140.57	149.04 ± 114.04	213.28 ± 174.59	< 0.001***
Sex, *n* (%)				
Male	542 (50.6%)	332 (46.6%)	210 (58.5%)	< 0.001***
Female	529 (49.4%)	380 (53.4%)	149 (41.5%)	
Race, *n* (%)				
Mexican American	458 (42.8%)	321 (45.1%)	137 (38.2%)	< 0.001***
Non‐Hispanic Black	375 (35.0%)	202 (28.4%)	173 (48.2%)	
Non‐Hispanic White	99 (9.2%)	78 (11.0%)	21 (5.8%)	
Other Hispanic	52 (4.9%)	36 (5.1%)	16 (4.5%)	
Other Race	87 (8.1%)	75 (10.5%)	12 (3.3%)	
Family PIR, *n* (%)				
≤ 1.0	270 (25.2%)	188 (26.4%)	82 (22.8%)	0.050*
> 1.0, ≤ 3.0	320 (29.9%)	223 (31.3%)	97 (27.0%)	
> 3.0	481 (44.9%)	301 (42.3%)	180 (50.1%)	
BMI, *n* (%)				
Underweight	6 (0.6%)	2 (0.3%)	4 (1.1%)	< 0.001***
Normal	119 (11.1%)	65 (9.1%)	54 (15.0%)	
Overweight	320 (29.9%)	192 (27.0%)	128 (35.7%)	
Obese	626 (58.5%)	453 (63.6%)	173 (48.2%)	
Age group, *n* (%)				
18–65	741 (69.2%)	643 (90.3%)	98 (27.3%)	< 0.001***
≥ 65	330 (30.8%)	69 (9.7%)	261 (72.7%)	
Education, *n* (%)				
Below high school	501 (46.8%)	299 (42.0%)	202 (56.3%)	< 0.001***
High school graduate/above	570 (53.2%)	413 (58.0%)	157 (43.7%)	
Marital status, *n* (%)				
Married/living with partner	686 (64.1%)	454 (63.8%)	232 (64.6%)	0.788
Widowed/separated/divorced/never married	385 (35.9%)	258 (36.2%)	127 (35.4%)	
Folate, ng/mL RBC (mean ± SD)	398.69 ± 211.70	426.63 ± 225.42	343.26 ± 168.51	< 0.001***
Vitamin B12, pg/mL (mean ± SD)	780.86 ± 6184.11	882.44 ± 7568.00	579.41 ± 701.16	0.290
Energy intake, kcal/day (mean ± SD)	1815.86 ± 844.64	1902.23 ± 873.07	1644.57 ± 757.74	< 0.001***
Protein intake, g/day (mean ± SD)	71.89 ± 38.44	75.38 ± 40.83	64.97 ± 32.14	< 0.001***
Smoking, *n* (%)				
Current smoker	164 (15.3%)	106 (14.9%)	58 (16.2%)	< 0.001***
Former smoker	303 (28.3%)	170 (23.9%)	133 (37.0%)	
Never smoker	604 (56.4%)	436 (61.2%)	168 (46.8%)	
Alcohol, *n* (%)				
Current drinker	667 (62.3%)	439 (61.7%)	228 (63.5%)	0.057
Former drinker	161 (15.0%)	98 (13.8%)	63 (17.5%)	
Lifetime abstainer	243 (22.7%)	175 (24.6%)	68 (18.9%)	
Physical activity, *n* (%)				
Inactive	410 (38.3%)	219 (30.8%)	191 (53.2%)	< 0.001***
Moderate	594 (55.5%)	427 (60.0%)	167 (46.5%)	
Vigorous	67 (6.3%)	66 (9.3%)	1 (0.3%)	
Congestive heart failure, *n* (%)				
No	1017 (95.0%)	698 (98.0%)	319 (88.9%)	< 0.001***
Yes	54 (5.0%)	14 (2.0%)	40 (11.1%)	
Diabetes, *n* (%)				
No	871 (81.3%)	597 (83.8%)	274 (76.3%)	0.006**
Yes	170 (15.9%)	95 (13.3%)	75 (20.9%)	
Borderline	30 (2.8%)	20 (2.8%)	10 (2.8%)	
Follow up, months (mean ± SD)	137.25 ± 75.85	149.10 ± 77.45	113.77 ± 66.69	< 0.001***

Abbreviations: BMI, body mass index; MMA, methylmalonic acid; PIR, poverty income ratio.

* *p* < 0.05.

** *p* < 0.01.

*** *p* < 0.001.

### 3.2 Association of MMA With Low Muscle Mass in the Weighted Logistic Regression

**FIGURE 2:**
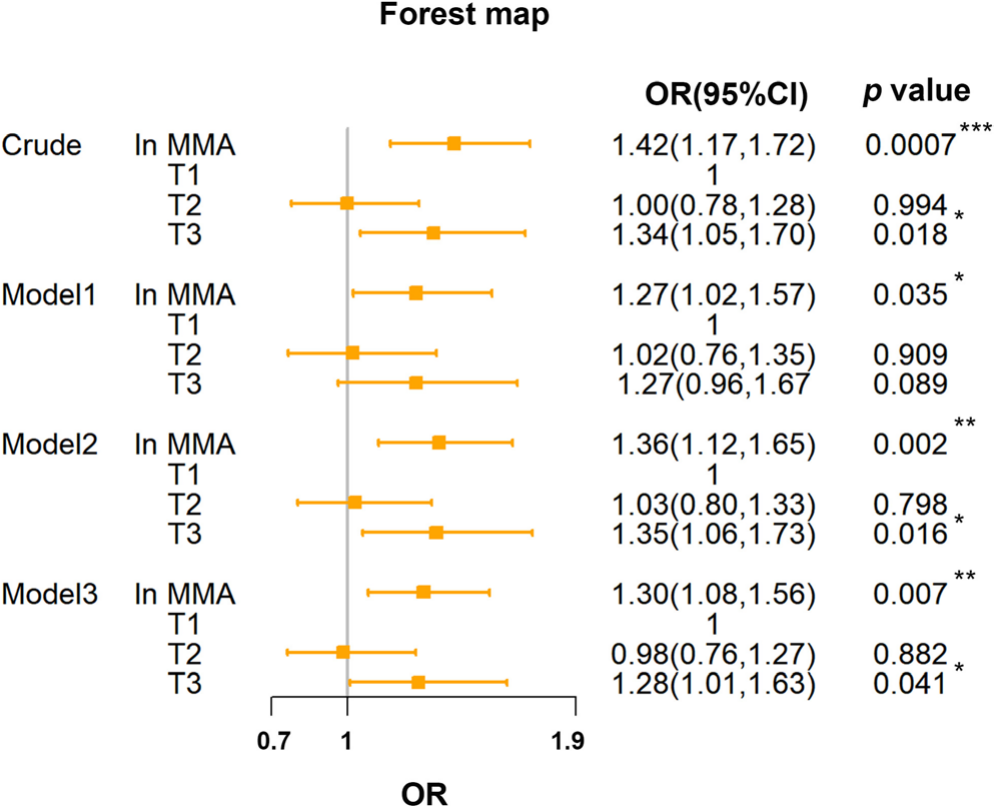
The associations between ln MMA and low muscle mass. Crude model (no covariates were adjusted); Model 1 (demographic data were adjusted); Model 2 (demographic and lifestyle data were adjusted); Model 3 (demographic data, lifestyle, and comorbidities were adjusted). *p < 0.05, **p < 0.01, ***p < 0.001.

We utilized a weighted multivariable logistic regression model to investigate the relationship between MMA and low muscle mass. It revealed a significant association between serum MMA level and low muscle mass prevalence (Figure 2). In the crude model, elevated MMA demonstrated a strong positive correlation with low muscle mass (OR = 1.42, 95% CI: 1.17–1.72, *p* = 0.0007). Sequential adjustments progressively attenuated this relationship while maintaining statistical significance. Model 1 (demographic covariates: age, gender, race, BMI, marital status, education, and family PIR) yielded an OR of 1.27 (95% CI: 1.02–1.57, *p* = 0.035). Model 2 (additional lifestyle adjustments: smoking/alcohol consumption, physical activity and dietary patterns) strengthened the association to OR = 1.36 (95% CI: 1.1–1.65, *p* = 0.002) and Model 3 (full adjustment including comorbidities such as congestive heart failure and diabetes) showed a 30% increased low muscle mass risk per natural log‐transformed MMA unit (ln MMA) (OR = 1.30, 95% CI: 1.08–1.56, *p* = 0.007). Categorical analysis of ln MMA tertiles further supported this dose–response pattern, with the highest tertile exhibiting 28% greater low muscle mass prevalence compared to the lowest tertile (Model 3: OR = 1.28, 95% CI: 1.01–1.63, *p* = 0.041). The consistent trend across models (*p* < 0.05) suggested MMA elevation independently contributed to low muscle mass pathophysiology.

### 3.3 MMA‐Mortality Association in Sarcopenia Adults: Weighted Cox Regression, RCS and Kaplan–Meier Survival Analysis

**FIGURE 3:**
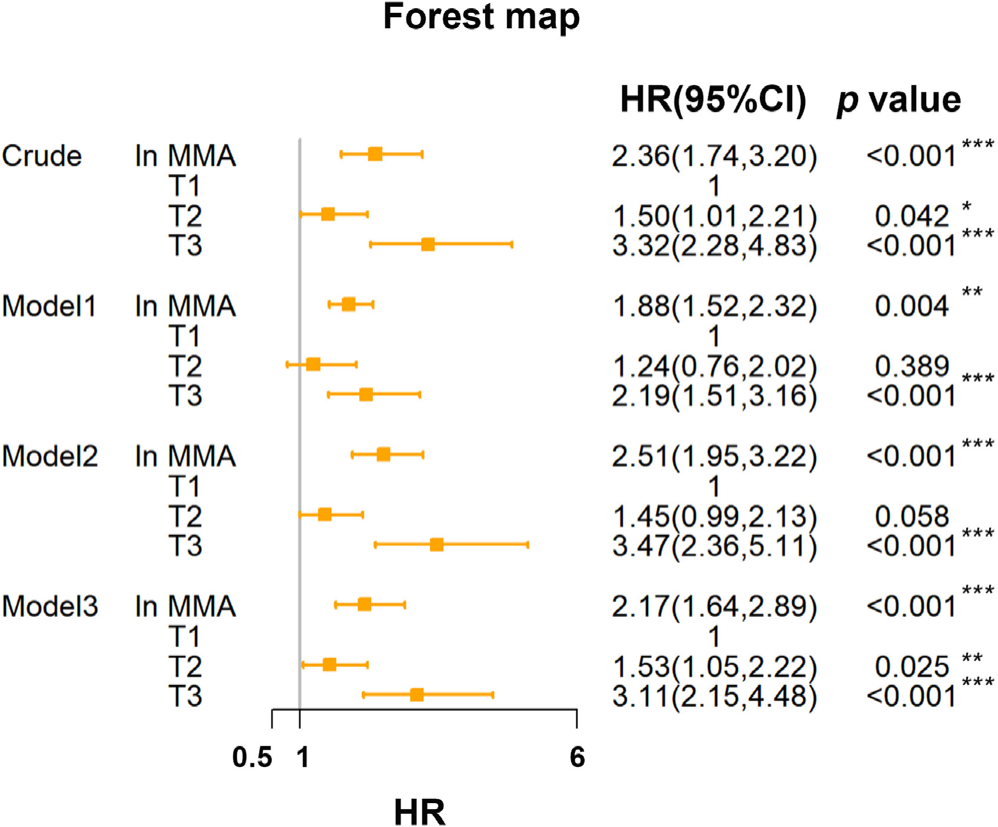
The associations between MMA and all‐cause mortality in low muscle mass adults. Crude model (no covariates were adjusted); Model 1 (demographic data were adjusted); Model 2 (demographic and lifestyle data were adjusted); Model 3 (demographic data, lifestyle, and comorbidities were adjusted). *p < 0.05, **p < 0.01, ***p < 0.001.

To further investigate whether serum MMA level was associated with all‐cause mortality in low muscle mass adults, we performed a weighted multivariate Cox regression analysis (Figure 3). It demonstrated significant mortality risks associated with MMA elevation in low muscle mass adults. Each standard deviation increase in ln MMA conferred 136% higher mortality risk (Crude model: HR = 2.36, 95% CI: 1.74–3.20, *p* < 0.001), retaining significance in Model 1 (HR = 1.88, 95% CI: 1.52–2.32, *p* = 0.004), Model 2 (HR = 2.51, 95% CI: 1.95–3.22, *p* < 0.001) and Model 3 (HR = 2.17, 95% CI: 1.64–2.89, *p* < 0.001). Simultaneously, the highest ln MMA tertile (T3) exhibited substantially elevated all‐cause mortality versus the reference tertile (Crude HR = 3.32, 95% CI: 2.28–4.83, *p* < 0.001). This association persisted through sequential adjustments: Model 1 maintained HR = 2.19 (95% CI: 1.51–3.16, *p* < 0.001), Model 2 showed HR = 3.47 (95% CI: 2.36–5.11, *p* < 0.001) and fully adjusted Model 3 confirmed 211% increased mortality risk (HR = 3.11, 95% CI: 2.15–4.48, *p* < 0.001). The concordant trends across both analytical approaches (*p* < 0.05 for all models) substantiated MMA as an independent prognostic biomarker, with particular clinical relevance observed at supranormal concentrations. Please refer to Figure 3 for detailed information.

**FIGURE 4:**
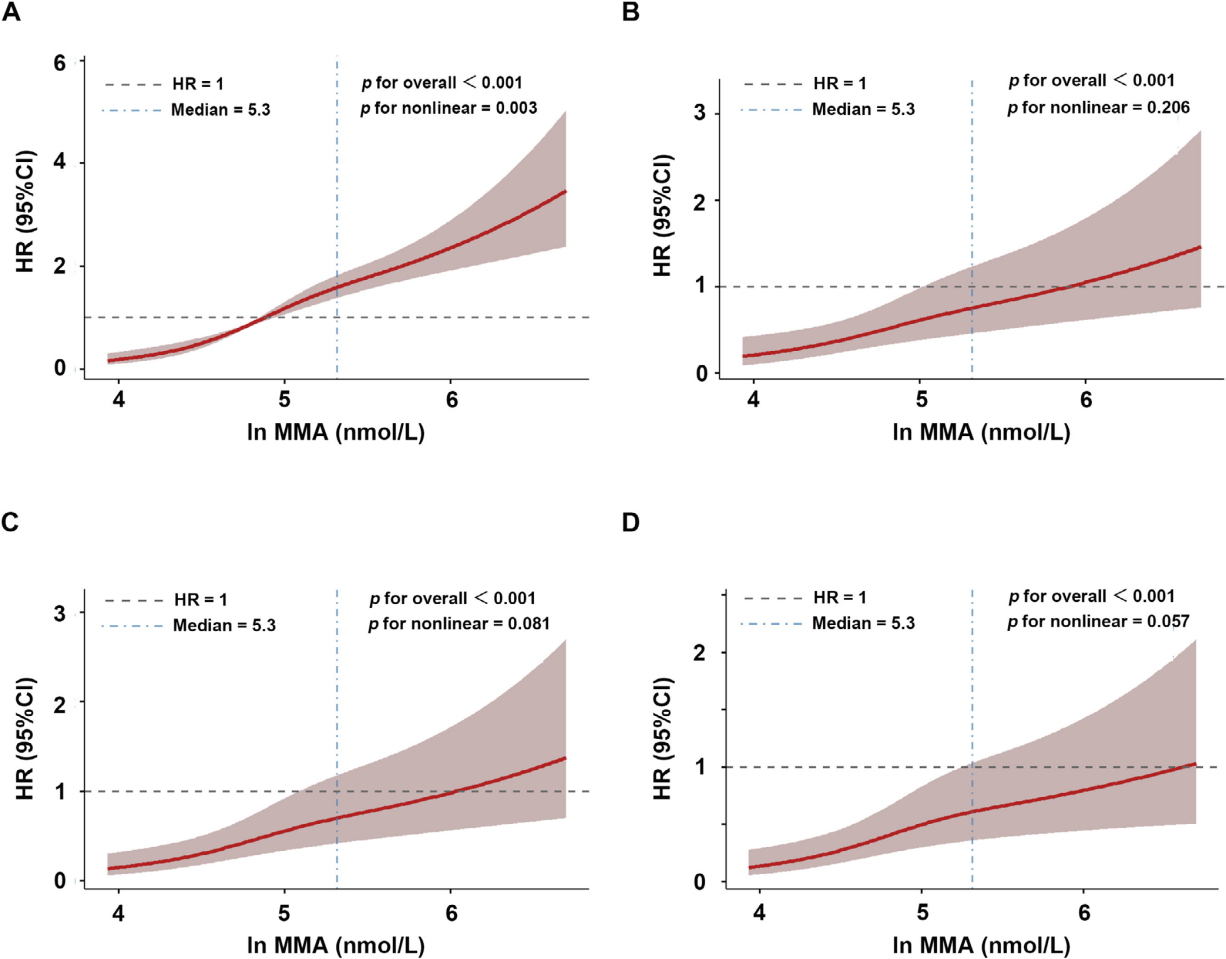
Weighted RCS curve of the relationship between MMA and mortality in low muscle mass adults: (A) no covariates were adjusted; (B) demographic data were adjusted; (C) demographic and lifestyle data were adjusted; (D) demographic data, lifestyle, and comorbidities were adjusted.

To further characterize the dose–response relationship, we implemented weighted restricted cubic splines (RCS) analysis to model mortality risk associated with ln MMA in low muscle mass adults (Figure 4). Initial unadjusted spline regression revealed a monotonic elevation in mortality hazard ratio (HR) with increasing MMA level (Figure 4A, *p* for overall < 0.001 and *p* for nonlinear = 0.003), suggesting threshold‐dependent effects at lower concentrations. Subsequent multivariable adjustments confirmed a persistent linear association (Figure 4B–D). All adjusted models demonstrated significant overall trends (*p* for overall < 0.001) but non‐significant nonlinear components (*p* for nonlinear > 0.05), indicating MMA‐mortality proportionality across biological concentration ranges. This coherence between categorical and continuous analytical paradigms reinforced MMA's role as a graded risk indicator rather than a threshold‐dependent biomarker.

**FIGURE 5:**
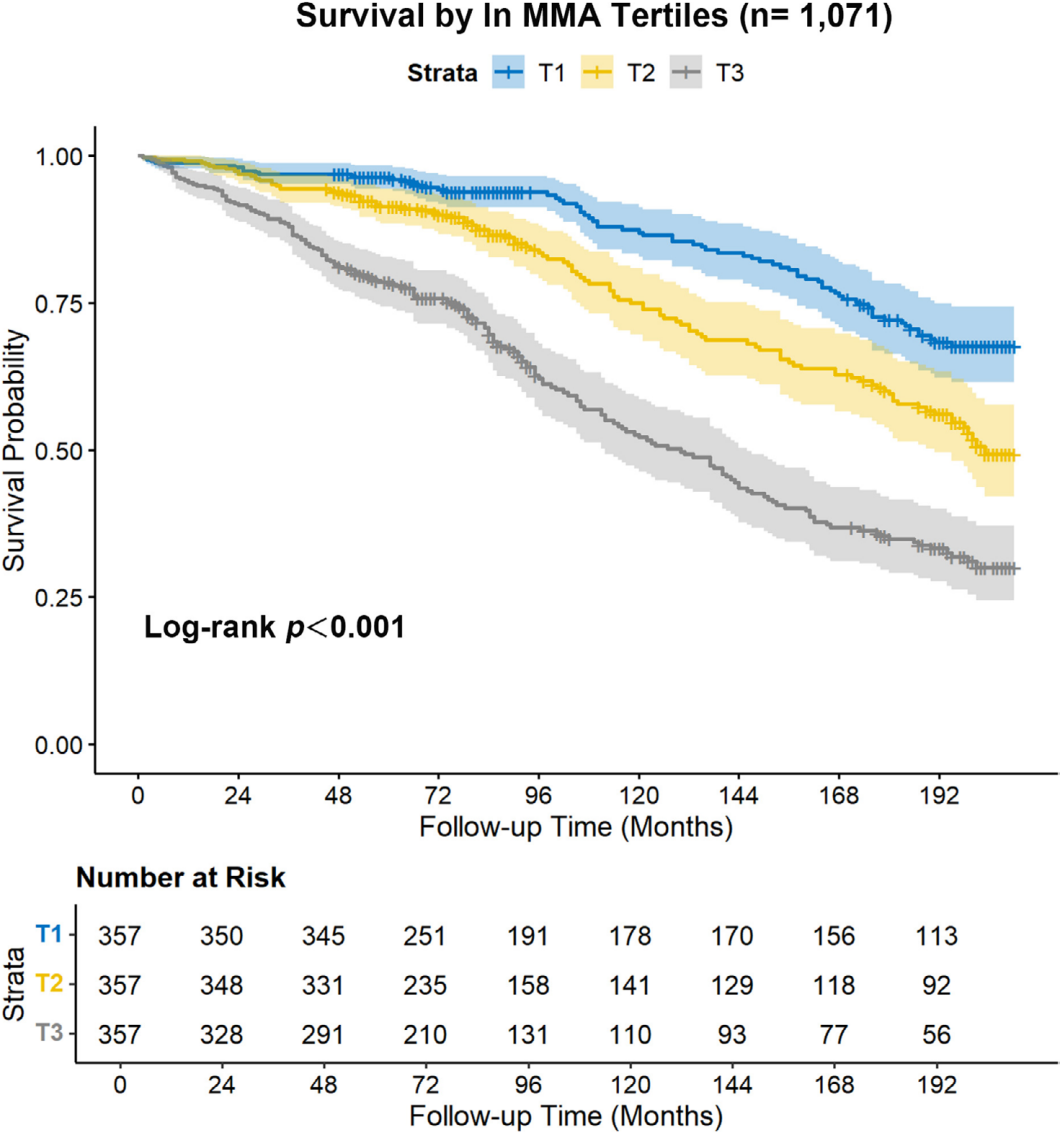
Kaplan–Meier survival curve on MMA and all‐cause mortality in low muscle mass adults.

Kaplan–Meier survival analysis stratified by tertiles of ln MMA revealed dose‐dependent mortality risk in low muscle mass adults (Figure 5). The log‐rank test confirmed significant survival disparities across ln MMA tertiles (*p* < 0.001), with the highest ln MMA tertile (T3) demonstrating markedly reduced survival probability relative to T1. Notably, when further stratifying the low muscle mass cohort by age, MMA level lost predictive power for survival outcomes in younger and middle‐aged groups (Log‐rank *p* > 0.05; Figure S2A,B), while maintaining robust prognostic significance in the ≥ 60‐year subgroup (Log‐rank *p* = 0.006; Figure S2C).

### 3.4 Sensitivity Analysis

Three separate weighted sensitivity analyses were carried out in order to confirm the stability and coherence of the study findings, as shown in Figure S1. First, after excluding participants with implausibly low energy intake (< 1000 kcal/day), 919 individuals remained. Following full adjustment for covariates, the positive correlation between ln MMA and mortality in low muscle mass adults remained stable (HR = 2.16, 95% CI: 1.61–2.89, *p* < 0.001). Second, the analysis excluding participants ≥ 65 years old (741 individuals remained) demonstrated a 166% risk elevation in T3 versus T1 (Model 3 HR = 2.66, 95% CI: 1.26–5.61, *p* = 0.010). Finally, exclusion of congestive heart failure participants (1017 individuals remained) preserved significant correlation (HR = 2.68, 95% CI: 2.19–3.27, *p* < 0.001). All sensitivity‐adjusted estimates retained statistical significance (*p* < 0.05), confirming outcome stability across clinically relevant subgroups.

### 3.5 Subgroup Analysis and Interaction

**FIGURE 6:**
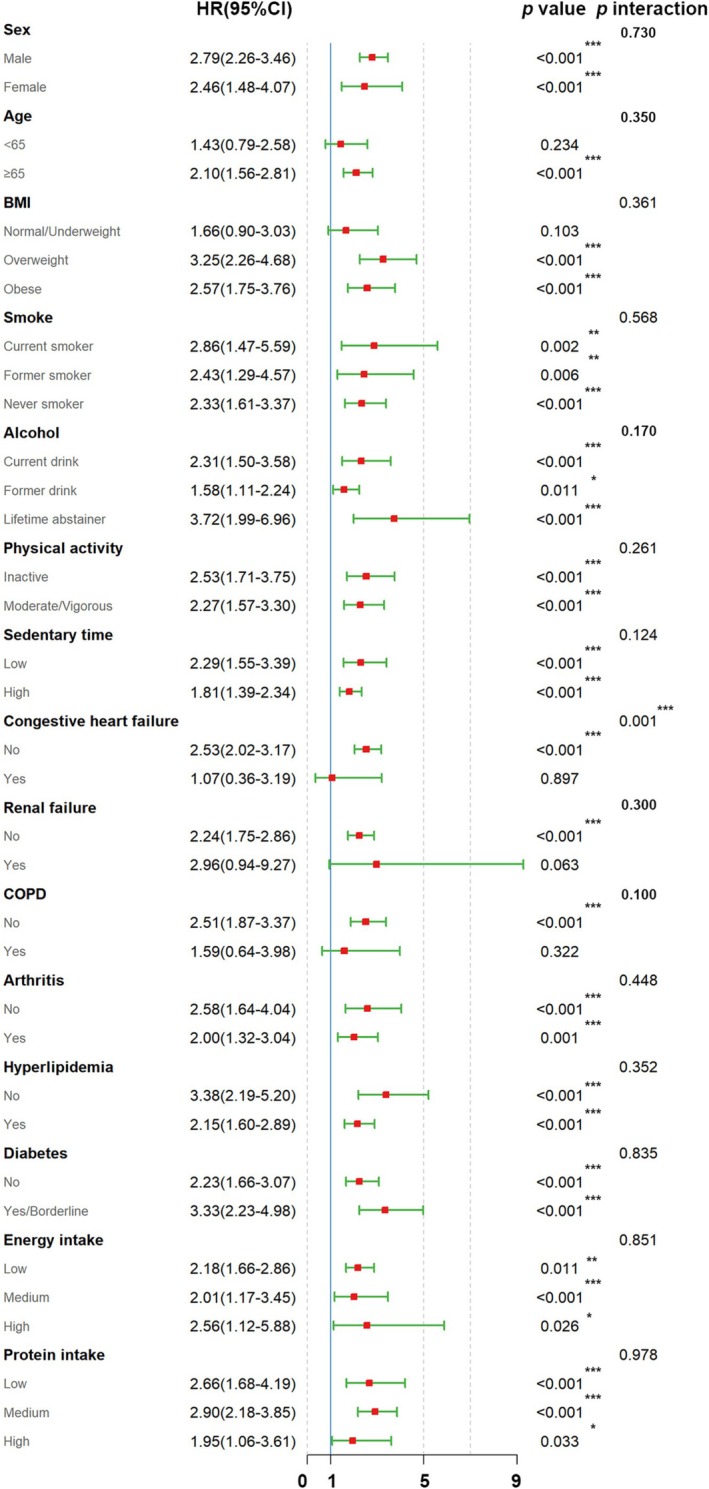
Subgroup analysis of the association of MMA and mortality of low muscle mass adults. COPD, chronic obstructive pulmonary disease.

Subgroup analysis across gender, age, BMI, smoking/drinking status, physical activity, sedentary time, nutrition‐related issues (energy/protein intake) and disease‐related causes (including congestive heart/renal failure, COPD, arthritis, hyperlipidemia and diabetes) demonstrated consistent positive MMA‐mortality associations in most of the low muscle mass population after controlling for all confounding factors. Notably, elevated risks emerged remarkably among lifetime alcohol abstainers (HR = 3.60, 95% CI: 2.34–5.53, *p* < 0.001) and diabetic/borderline patients (HR = 3.49, 95% CI: 2.35–5.20, *p* < 0.001). Additionally, no significant interactions were identified in any subgroups, with the exception of the congestive heart failure subgroup (*p* for interaction = 0.001), confirming MMA's deleterious prognostic impact across biological and behavioral strata. These subgroup‐specific dose–response patterns substantiate MMA as an independent mortality predictor in low muscle mass pathophysiology (Figure 6).

## 4 Discussion

While increasing insights have highlighted mitochondrial dysfunction and oxidative stress as pivotal contributors to sarcopenia pathogenesis, progression, and prognosis, human observational studies directly linking mitochondrial biomarkers to sarcopenia incidence and outcomes have been limited (Habets et al. 2022; Kim et al. 2023; Li et al. 2022; Xu et al. 2025; Zhang et al. 2023). Our population‐based longitudinal study of 10,414 U.S. adults addressed this gap by evaluating serum MMA, a mitochondria‐derived metabolite, in low muscle mass risk and prognosis.

Weighted multivariable analysis robustly associated elevated MMA level with low muscle mass incidence, supporting its role as a population‐level predictor. The positive association between serum MMA and sarcopenia was consistent with previous studies linking mitochondrial dysfunction metabolites to muscle atrophy (Kemp et al. 2020; Kunzke et al. 2020; Marques et al. 2023; Sha et al. 2023). Additionally, the association between MMA level and mortality has already been investigated in general and disease‐specific cohorts (Guo et al. 2023; Liu et al. 2024; Wang, Tang, et al. 2022; Wang et al. 2020; Wu et al. 2023; Zhan et al. 2024). Our study extended prior evidence by demonstrating MMA also remained a critical prognostic insight even after sarcopenia onset, especially in the elder population. Among adults of low muscle mass, weighted Cox models demonstrated the MMA and mortality association was independent of confounders, with RCS analysis confirming a dose‐dependent risk escalation. Furthermore, subgroup analysis revealed that compared to secondary sarcopenia caused by various diseases such as metabolic disorders and arthritis, MMA held a more unique predictive value for the prognosis of age‐related primary sarcopenia. A significant interaction emerged between MMA and congestive heart failure (CHF). It likely reflected statistical instability from limited sample size (*n* for CHF = 54/1071, 5% of cohort) rather than a biological null effect, necessitating validation in larger cohorts.

The positive association between serum MMA and mortality in sarcopenic adults likely arose through mitochondrial dysfunction and oxidative stress, then amplified the metabolic vulnerability of weak muscle. As a toxic mitochondrial metabolite, MMA disrupted energy metabolism by suppressing the TCA cycle, impairing ATP synthesis, and inhibiting fatty acid β‐oxidation, thereby exacerbating the “energy crisis” (Green and Miller 2022; Cao et al. 2024; Chandler and Venditti 2019; Guo et al. 2024; Liu et al. 2022; Zhan et al. 2024). Therefore, mitochondria‐rich tissues (such as brain, heart, kidney, and skeletal muscle) would be seriously damaged, leading to multi‐organ failure and adverse outcomes (Manoli et al. 2013; Ostergaard et al. 2007; Stepien et al. 2017; Wolffenbuttel et al. 2020; Zhao et al. 2024). Concurrently, MMA drove oxidative stress via: (1) ROS overproduction from Complex II–III inhibition, (2) glutathione depletion through downregulating solute carrier family 7 member 11 (SLC7A11)/glutathione peroxidase 4 (GPX4) expression, and (3) lactate accumulation by blocking Lactate dehydrogenase (LDH)‐mediated (Proctor et al. 2020; Wongkittichote et al. 2019; Chu et al. 2019; Weidemann et al. 1970; Guo et al. 2024; Li et al. 2021). These effects collectively overwhelmed antioxidant defenses, causing lipid/protein oxidation and mtDNA damage, which further aggravated mitochondrial dysfunction in a self‐perpetuating loop. Sustained oxidative stress also activated inflammatory cascades and accelerated muscle wasting, a cachexia state linked to poor prognosis (de Souza Almeida et al. 2022; Ganie et al. 2024; Li et al. 2025). Corroboratively, our subgroup analysis also revealed that the positive association between serum MMA and mortality was more pronounced in diabetic individuals and lifetime alcohol abstainers. In diabetes, pre‐existing metabolic disturbances may synergize with MMA‐induced mitochondrial dysfunction and ROS overproduction (Black 2022; Charlton et al. 2020; Li et al. 2024). Abstainers lacked alcohol‐adaptive upregulation of glutamate‐cysteine ligase, rendering them vulnerable to acute ROS surges and GSH depletion under MMA challenge (Bloomer et al. 2021; Chen et al. 2019). Thus, MMA served as a metabolic disruptor that converges mitochondrial dysfunction and oxidative stress to drive mortality in sarcopenic adults.

Methodologically, our findings withstand scrutiny through multiple strengths: a nationally representative cohort, standardized sarcopenia diagnostic criteria (FNIH), comprehensive adjustment for demographic data, lifestyle, and comorbidities, and replication via multiple analytical approaches. The consistent results across sensitivity analyses mitigate concerns about the robustness of the results. Although residual confounding from unmeasured factors (e.g., genetic polymorphisms in MMA metabolism and other comorbid factors) remains possible. Clinically, these data suggest serum MMA could serve as a dual‐purpose biomarker for both sarcopenia screening and mortality prediction in diagnosed cases to enable early‐stage risk stratification and precise prediction of adverse outcomes. However, using serum MMA level to guide treatment faces practical hurdles. First, the test costs high. Second, serum B12 level often do not reliably show if the body is actually using it. Also, giving high‐dose B12 injections to frail elderly individuals without clear proof of deficiency might cause extra discomfort or risks. Therefore, for future sarcopenia management, we should explore more appropriate ways to lower MMA level and carefully add B12 supplements only after confirming actual deficiency (She et al. 2025; Sonntag et al. 2022). Future clinical trials will be needed to confirm if these strategies work.

While leveraging NHANES' rigorous protocols, our study had limitations: (1) Cross‐sectional sarcopenia assessment precluded causal inference for disease onset. (2) Single MMA measurement may underestimate chronic exposure effects. (3) Given that sarcopenia encompasses both muscle loss and functional decline, our reliance on muscle mass reduction alone as the primary measure might not fully encapsulate the functional dimensions of the condition. (4) Lack of muscle biopsy data limited mechanistic exploration. Future research should focus on exploring the effects of prospective cohorts with repeated MMA measurements and multi‐omics profiling to clarify temporal relationships and molecular pathways.

## 5 Conclusion

This study established that serum methylmalonic acid (MMA) could be a dual biomarker for early sarcopenia prediction and prognosis. Further research is needed to explore the causal relationship between MMA and sarcopenia or mortality in sarcopenic adults, as well as to elucidate the underlying mechanisms involved. Additionally, targeted interventions including B12 supplementation, mitochondrial enhancers, and dietary modulators of MMA metabolism warrant validation in randomized trials to determine their efficacy in halting sarcopenia progression and reducing mortality in high‐risk populations.

## Author Contributions

**Ping Zhu:** investigation (equal), methodology (equal), software (equal), visualization (equal), writing – original draft (equal). **Jie Zhang:** supervision (equal). **Xue‐Chun Liu:** supervision (equal). **Ming Song:** funding acquisition (equal), supervision (equal), writing – review and editing (equal). **Bin Lu:** supervision (equal), writing – review and editing (equal). **Zhi‐Cheng Yang:** conceptualization (equal), resources (equal), software (equal), supervision (equal), validation (equal), visualization (equal). **Hui Pan:** data curation (equal), project administration (equal). **Ya‐Qiong Jiao:** data curation (equal), project administration (equal). **Ya‐Fei Guo:** data curation (equal), project administration (equal). **Fang‐Fang Chen:** funding acquisition (equal), supervision (equal). **Zhi‐Hao Wang:** funding acquisition (equal), supervision (equal). **Bo‐Ang Hu:** conceptualization (equal), formal analysis (equal), project administration (equal), supervision (equal), writing – review and editing (equal). **Ming Zhong:** supervision (equal).

## Ethics Statement

The studies involving humans were approved by the National Center for Health Statistics Ethics Review Board. The studies were conducted in accordance with local legislation and institutional requirements. The participants provided their written informed consent to participate in this study.

## Conflicts of Interest

The authors declared that the research was conducted in the absence of any commercial or financial relationships that could be construed as a potential conflicts of interest.

## Supporting information


**Figure S1:** fsn370841‐sup‐0001‐FigureS1.docx.

**Table S1:** fsn370841‐sup‐0002‐TableS1.docx.

## Data Availability

Publicly available datasets were analyzed in this study. The datasets could be downloaded for free from the website: https://www.cdc.gov/nchs/nhanes/index.htm.
