# Supplementary material for: The Role of Methylmalonic Acid in the Risk of Sarcopenia and All‐Cause Mortality Among Individuals With Sarcopenia: Evidence From NHANES
Source: Food Sci Nutr. 2025 Sep 3;13(9):e70841. doi: 10.1002/fsn3.70841 (PMC12406174; doi:10.1002/fsn3.70841)
Supplement: Supplementary file 1 — Figure S1: fsn370841‐sup‐0001‐FigureS1.docx. [file FSN3-13-e70841-s002.docx]

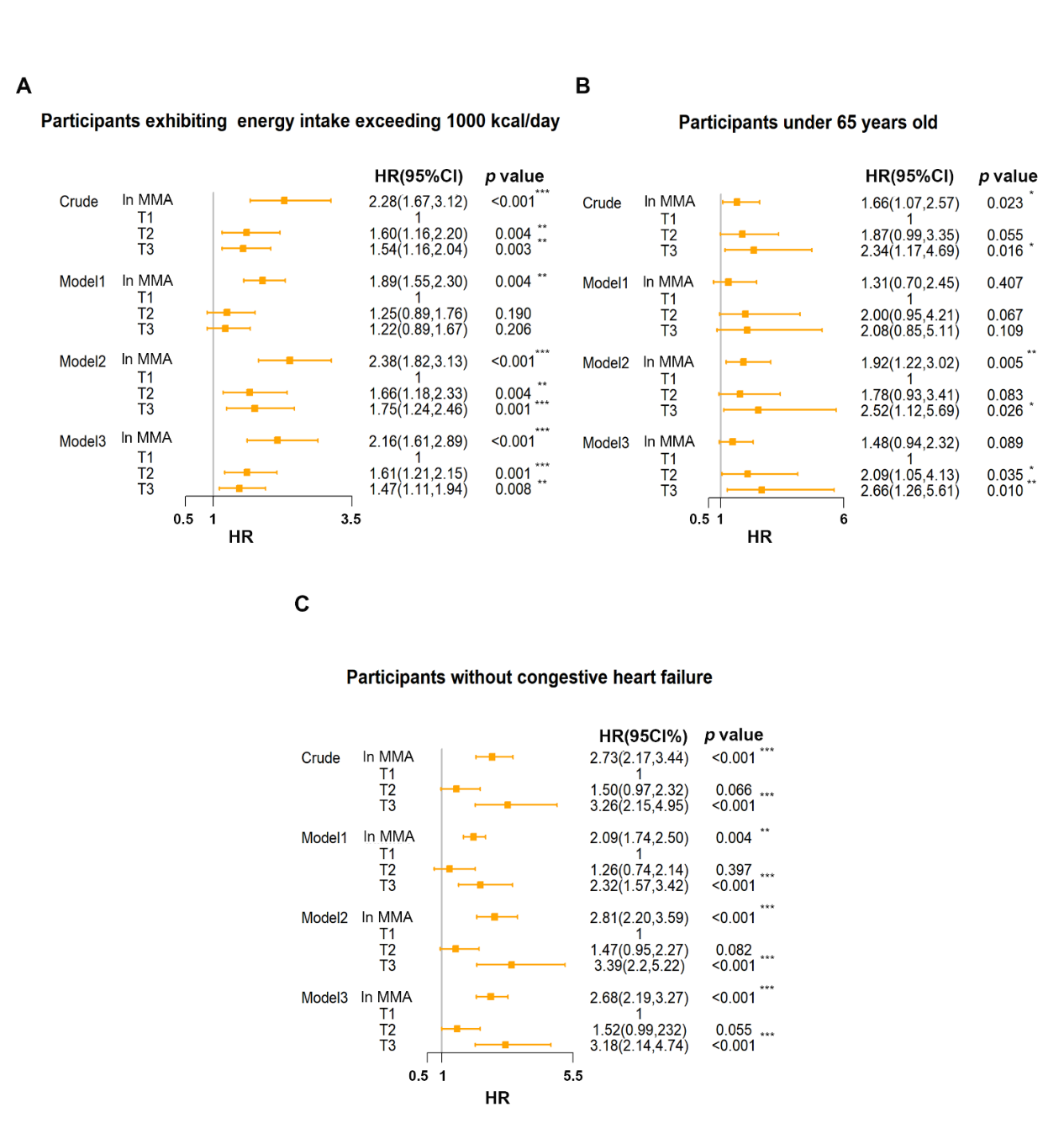


**Supplementary Figure.1** **The relationship between MMA and mortality in low muscle mass adults**

(A) Participants whose energy intake <1000 kcal/day were deleted; (B) Participants aged ≥ 65 years old were deleted; (C) Participants with congestive heart failure were deleted. Crude Model (no covariates were adjusted); Model1 (demographic data were adjusted); Model2 (demographic and lifestyle data were adjusted); Model3 (demographic data, lifestyle and comorbid comorbidities were adjusted).


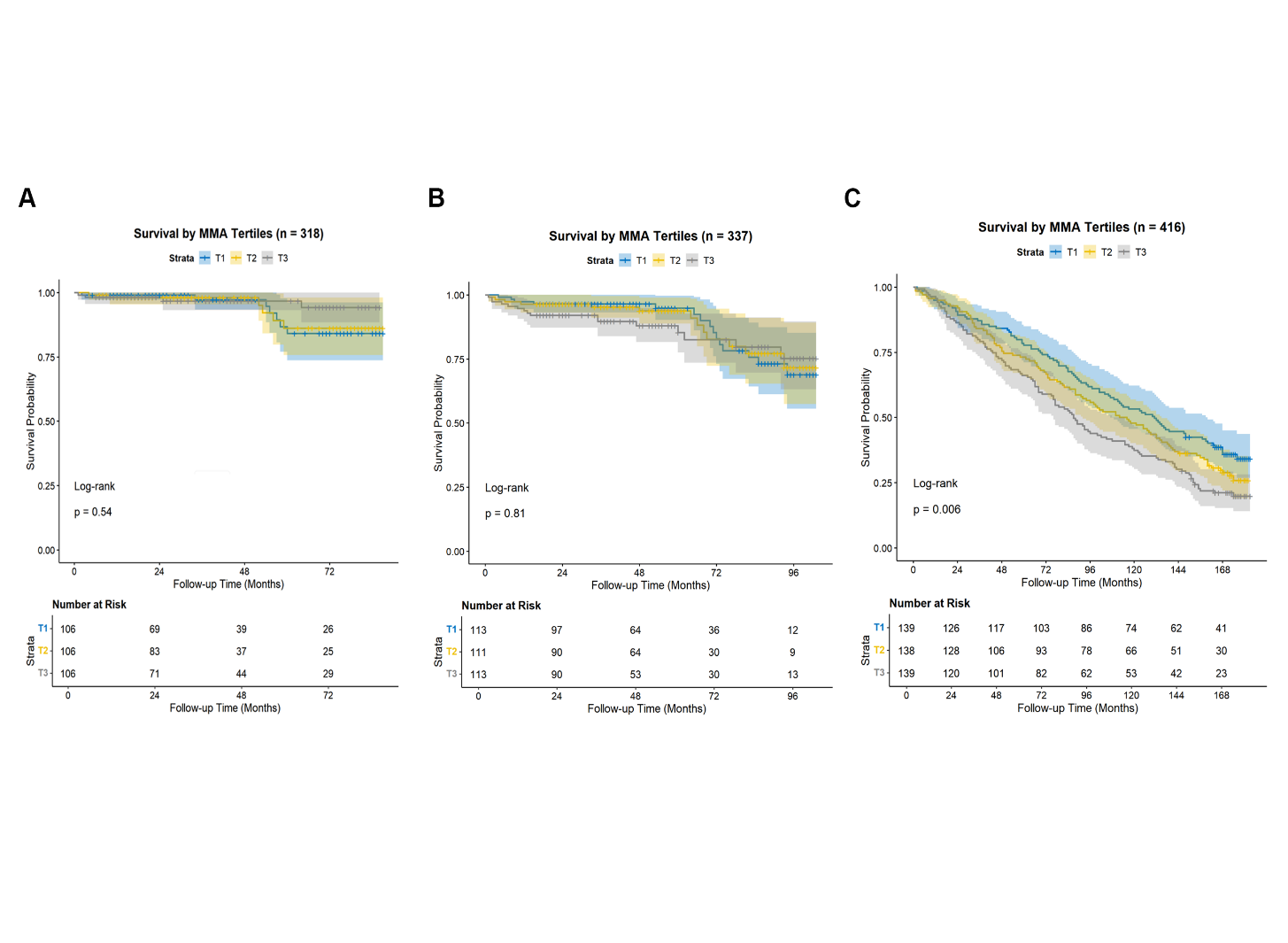


**Supplementary Figure. 2 Age-stratified relationship between MMA and mortality in adults with low muscle mass**

(A) Kaplan-Meier survival curves on MMA and all-cause mortality in low muscle mass adults aged 18-45 years. (B) Kaplan-Meier survival curves on MMA and all-cause mortality in low muscle mass adults aged 45-60 years. (C) Kaplan-Meier survival curves on MMA and all-cause mortality in low muscle mass adults aged≥60 years.
