# Supplementary material for: The Role of Methylmalonic Acid in the Risk of Sarcopenia and All‐Cause Mortality Among Individuals With Sarcopenia: Evidence From NHANES
Source: Food Sci Nutr. 2025 Sep 3;13(9):e70841. doi: 10.1002/fsn3.70841 (PMC12406174; doi:10.1002/fsn3.70841)
Supplement: Supplementary file 2 — Table S1: fsn370841‐sup‐0002‐TableS1.docx. [file FSN3-13-e70841-s001.docx]

| **Characteristics** | **Overall**  (N = 462,911,472.766465) | **Normal muscle mass** (N = 462,911,472.766465) | **Low muscle mass**  (N = 462,911,472.766465) | **P-value** |
| --- | --- | --- | --- | --- |
| **MMA, nmol/L (mean±SD)** | 153.06 ± 104.62 | 148.24 ± 99.45 | 170.57 ± 140.57 | <0.001 |
| **Sex, n (%)** |  |  |  | 0.225 |
| Male | 233,482,130 (50.4%) | 215,259,255 (50.2%) | 18,222,876 (52.8%) |  |
| Female | 229,429,343 (49.6%) | 213,169,977 (49.8%) | 16,259,366 (47.2%) |  |
| **Race, n (%)** |  |  |  | <0.001 |
| Mexican American | 38,982,654 (8.4%) | 32,433,717 (7.6%) | 6,548,937 (19.0%) |  |
| Non-Hispanic Black | 308,815,431 (66.7%) | 287,993,190 (67.2%) | 20,822,241 (60.4%) |  |
| Non-Hispanic White | 33,501,313 (7.2%) | 30,017,691 (7.0%) | 3,483,622 (10.1%) |  |
| Other Hispanic | 50,251,266 (10.9%) | 49,225,490 (11.5%) | 1,025,776 (3.0%) |  |
| Other Race | 31,360,808 (6.8%) | 28,759,144 (6.7%) | 2,601,665 (7.5%) |  |
| **Family PIR, n (%)** |  |  |  | <0.001 |
| ≤1.0 | 79,811,180 (17.2%) | 72,084,112 (16.8%) | 7,727,068 (22.4%) |  |
| >1.0, ≤3.0 | 163,776,615 (35.4%) | 148,686,835 (34.7%) | 15,089,780 (43.8%) |  |
| >3.0 | 219,323,677 (47.4%) | 207,658,284 (48.5%) | 11,665,393 (33.8%) |  |
| **BMI, n (%)** |  |  |  | <0.001 |
| Underweight | 8,270,108 (1.8%) | 8,041,219 (1.9%) | 228,890 (0.7%) |  |
| Normal | 148,240,634 (32.0%) | 145,223,948 (33.9%) | 3,016,686 (8.7%) |  |
| Overweight | 155,622,874 (33.6%) | 146,829,112 (34.3%) | 8,793,762 (25.5%) |  |
| Obese | 150,777,857 (32.6%) | 128,334,953 (30.0%) | 22,442,904 (65.1%) |  |
| **Age group, n (%)** |  |  |  | <0.001 |
| 18-65 | 433,465,767 (93.6%) | 406,169,028 (94.8%) | 27,296,739 (79.2%) |  |
| ≥65 | 29,445,706 (6.4%) | 22,260,204 (5.2%) | 7,185,502 (20.8%) |  |
| **Education, n (%)** |  |  |  | <0.001 |
| Below high school | 84,085,564 (18.2%) | 73,304,721 (17.1%) | 10,780,843 (31.3%) |  |
| High school graduate/above | 378,825,909 (81.8%) | 355,124,510 (82.9%) | 23,701,399 (68.7%) |  |
| **Marital status, n (%)** |  |  |  | <0.001 |
| Married/Living with partner | 282,662,296 (61.1%) | 260,567,157 (60.8%) | 22,095,139 (64.1%) |  |
| Widowed/Separated/  Divorced/Never married | 180,249,177 (38.9%) | 167,862,074 (39.2%) | 12,387,103 (35.9%) |  |
| **Folate, ng/mL RBC (mean±SD)** | 414.04 ± 201.54 | 412.04 ± 200.08) | 438.84 ± 217.45 | 0.008 |
| **Vitamin B12, pg/mL (mean±SD)** | 569.84 ± 1,647.32 | 568.39 ± 1,495.55) | 587.91 ± 2,940.61 | 0.673 |
| **Energy intake, kcal/day (mean±SD)** | 2,263.91 ± 1,045.83 | 2,290.58 ± 1,054.23) | 1,932.54 ± 869.94 | <0.001 |
| **Protein intake, g/day (mean±SD)** | 85.07 ± 44.46 | 85.88 ± 44.80) | 75.07 ± 38.75 | <0.001 |
| **Smoke, n (%)** |  |  |  | <0.001 |
| Current smoker | 109,819,651 (23.7%) | 103,786,106 (24.2%) | 6,033,545 (17.5%) |  |
| Former smoker | 97,818,765 (21.1%) | 88,757,334 (20.7%) | 9,061,431 (26.3%) |  |
| Never smoker | 255,273,057 (55.1%) | 235,885,792 (55.1%) | 19,387,266 (56.2%) |  |
| **Alcohol, n (%)** |  |  |  | <0.001 |
| Current drinker | 358,568,306 (77.5%) | 335,668,773 (78.3%) | 22,899,533 (66.4%) |  |
| Former drinker | 51,827,433 (11.2%) | 47,447,255 (11.1%) | 4,380,178 (12.7%) |  |
| Lifetime abstainer | 52,515,734 (11.3%) | 45,313,203 (10.6%) | 7,202,531 (20.9%) |  |
| **Physical activity, n (%)** |  |  |  | 0.002 |
| Inactive | 142,337,990 (30.7%) | 130,828,796 (30.5%) | 11,509,194 (33.4%) |  |
| Moderate | 267,691,757 (57.8%) | 247,194,266 (57.7%) | 20,497,491 (59.4%) |  |
| Vigorous | 52,881,726 (11.4%) | 50,406,169 (11.8%) | 2,475,557 (7.2%) |  |
| **Congestive heart failure, n (%)** |  |  |  | <0.001 |
| No | 456,766,119 (98.7%) | 423,540,214 (98.9%) | 343,565,905 (96.4%) |  |
| Yes | 6,145,354 (1.3%) | 4,889,017 (1.1%) | 1,256,337 (3.6%) |  |
| **Diabetes, n (%)** |  |  |  | <0.001 |
| No | 430,129,197 (92.9%) | 401,299,952 (93.7%) | 28,829,245 (83.6%) |  |
| Yes | 25,868,074 (5.6%) | 21,105,634 (4.9%) | 4,762,440 (13.8%) |  |
| Borderline | 6,914,202 (1.5%) | 6,023,645 (1.4%) | 890,557 (2.6%) |  |
| **Survival, n (%)** |  |  |  | <0.001 |
| No | 48,425,140 (10.5%) | 39,133,284 (9.1%) | 9.291,856 (26.9%) |  |
| Yes | 414,486,333 (89.5%) | 389,295,947 (90.9%) | 25,190,386 (73.1%) |  |
| **Follow up, months (mean±SD)** | 140.87 ± 74.53 | 142.04 ± 74.72) | 126.28 ± 70.58) | 0.002 |

**Supplementary Table 1: Weighted baseline characteristics of included participants according to muscle mass status. MMA, Methylmalonic acid; PIR, Poverty income ratio; BMI, body mass index.**

| **Characteristics** | **Overall** (N = 34,482,241.5113825) | **Alive** (N = 34,482,241.5113825) | **Deceased** (N = 34,482,241.5113825) | **P-value** |
| --- | --- | --- | --- | --- |
| **MMA, nmol/L (mean±SD)** | 169.23 ± 128.97 | 153.35 ± 109.95 | 212.27 ± 162.74 | <0.001 |
| **Sex, n (%)** |  |  |  | 0.295 |
| Male | 18,222,876 (52.8%) | 13,009,266 (51.6%) | 5,213,609 (56.1%) |  |
| Female | 16,259,366 (47.2%) | 12,181,120 (48.4%) | 4,078,246 (43.9%) |  |
| **Race, n (%)** |  |  |  | <0.001 |
| Mexican American | 6,548,937 (19.0%) | 5,925,123 (23.5%) | 623,814 (6.7%) |  |
| Non-Hispanic Black | 20,822,241 (60.4%) | 13,755,226 (54.6%) | 7,067,015 (76.1%) |  |
| Non-Hispanic White | 3,483,622 (10.1%) | 2,635,254 (10.5%) | 848,368 (9.1%) |  |
| Other Hispanic | 1,025,776 (3.0%) | 827,485 (3.3%) | 198,291 (2.1%) |  |
| Other Race | 2,601,665 (7.5%) | 2,047,297 (8.1%) | 554,368 (6.0%) |  |
| **Family PIR, n (%)** |  |  |  | 0.016 |
| ≤1.0 | 7,727,068 (22.4%) | 6,017,647 (23.9%) | 1,709,421 (18.4%) |  |
| >1.0, ≤3.0 | 15,089,780 (43.8%) | 10,249,305 (40.7%) | 4,840,475 (52.1%) |  |
| >3.0 | 11,665,393 (33.8%) | 8,923,434 (35.4%) | 2,741,960 (29.5%) |  |
| **BMI, n (%)** |  |  |  | <0.001 |
| Underweight/Normal | 3,245,575 (9.4%) | 1,784,708 (7.1%) | 1,460,868 (15.7%) |  |
| Overweight | 8,793,762 (25.5%) | 6,204,918 (24.6%) | 2,588,845 (27.9%) |  |
| Obese | 22,442,904 (65.1%) | 17,200,761 (68.3%) | 5,242,143 (56.4%) |  |
| **Age group, n (%)** |  |  |  | <0.001 |
| 18-65 | 27,296,739 (79.2%) | 23,910,039 (94.9%) | 3,386,700 (36.4%) |  |
| ≥65 | 7,185,502 (20.8%) | 1,280,347 (5.1%) | 5,905,155 (63.6%) |  |
| **Education, n (%)** |  |  |  | 0.003 |
| Below high school | 10,780,843 (31.3%) | 7,131,437 (28.3%) | 3,649,406 (39.3%) |  |
| High school graduate/above | 23,701,399 (68.7%) | 18,058,949 (71.7%) | 5,642,450 (60.7%) |  |
| **Marital status, n (%)** |  |  |  | 0.966 |
| Married/Living with partner | 22,095,139 (64.1%) | 16,127,812 (64.0%) | 5,967,327 (64.2%) |  |
| Widowed/Separated/  Divorced/Never married | 12,387,103 (35.9%) | 9,062,574 (36.0%) | 3,324,529 (35.8%) |  |
| **Folate, ng/mL RBC (mean±SD)** | 438.84 ± 217.45 | 462.78 ± 224.06 | 373.91 ± 183.62 | <0.001 |
| **Vitamin B12, pg/mL (mean±SD)** | 587.91 ± 2,940.61 | 611.74 ± 3,430.48 | 523.30 ± 442.39 | 0.206 |
| **Energy intake, kcal/day (mean±SD)** | 1,932.54 ± 869.94 | 1,996.83 ± 886.27 | 1,758.25 ± 799.76 | 0.011 |
| **Protein intake, g/day (mean±SD)** | 75.07 ± 38.75 | 77.43 ± 40.88 | 68.66 ± 31.47 | 0.008 |
| **Smoke, n (%)** |  |  |  | 0.020 |
| Current smoker | 6,033,545 (17.5%) | 3,945,738 (15.7%) | 2,087,807 (22.5%) |  |
| Former smoker | 9,061,431 (26.3%) | 6,227,597 (24.7%) | 2,833,834 (30.5%) |  |
| Never smoker | 19,387,266 (56.2%) | 15,017,051 (59.6%) | 4,370,215 (47.0%) |  |
| **Alcohol, n (%)** |  |  |  | 0.071 |
| Current drink | 22,899,533 (66.4%) | 17,031,890 (67.6%) | 5,867,643 (63.1%) |  |
| Former drink | 4,380,178 (12.7%) | 2,715,137 (10.8%) | 1,665,041 (17.9%) |  |
| Lifetime abstainer | 7,202,531 (20.9%) | 5,443,359 (21.6%) | 1,759,172 (18.9%) |  |
| **Physical activity, n (%)** |  |  |  | <0.001 |
| Inactive | 11,509,194 (33.4%) | 6,955,842 (27.6%) | 4,553,352 (49.0%) |  |
| Moderate | 20,497,491 (59.4%) | 15,805,444 (62.7%) | 4,692,046 (50.5%) |  |
| Vigorous | 2,475,557 (7.2%) | 2,429,100 (9.6%) | 46,457 (0.5%) |  |
| **Congestive heart failure, n (%)** |  |  |  | <0.001 |
| No | 33,225,905 (96.4%) | 24,711,519 (98.1%) | 8,514,386 (91.6%) |  |
| Yes | 1,256,337 (3.6%) | 478,867 (1.9%) | 777,469 (8.4%) |  |
| **Diabetes, n (%)** |  |  |  | 0.090 |
| No | 28,829,245 (83.6%) | 21,496,293 (85.3%) | 7,332,952 (78.9%) |  |
| Yes | 4,762,440 (13.8%) | 2,982,957 (11.8%) | 1,779,483 (19.2%) |  |
| Borderline | 890,557 (2.6%) | 711,136 (2.8%) | 179,421 (1.9%) |  |
| **Follow up, months (mean±SD)** | 126.28 ± 70.58 | 131.29 ± 70.94 | 112.68 ± 67.84 | 0.019 |

**Supplementary Table 2: Baseline characteristics of low muscle mass participants according to mortality status. MMA, Methylmalonic acid; PIR, Poverty income ratio; BMI, body mass index.**
